# Ferroptosis-mediated metabolic reprogramming as a driver of the inflammatory microenvironment in neutrophilic asthma: a perspective

**DOI:** 10.3389/fimmu.2026.1848122

**Published:** 2026-06-26

**Authors:** Mei-zhen Song, Xue-hui Wang

**Affiliations:** 1 Graduate School of Heilongjiang University of Chinese Medicine, Harbin, China; 2 Department of Respiratory Medicine, First Affiliated Hospital of Heilongjiang University of Chinese Medicine, Harbin, China

**Keywords:** ferroptosis, glucocorticoid resistance, lipid peroxidation, metabolic reprogramming, neutrophilic asthma

## Abstract

Neutrophilic asthma is a severe asthma subtype marked by persistent airway neutrophilia, Th17-skewed inflammation, and poor responsiveness to glucocorticoids. The mechanisms driving chronic neutrophilic inflammation and corticosteroid resistance remain incompletely understood. Ferroptosis is an iron-dependent form of regulated cell death driven by lipid peroxidation and redox imbalance. Emerging evidence suggests that ferroptosis is closely linked to cellular metabolism and immune regulation in inflammatory diseases. In this perspective article, we propose a hypothesis-generating framework in which ferroptosis-associated metabolic dysregulation may contribute to the development and persistence of neutrophilic asthma. Specifically, increased acyl-CoA synthetase long-chain family member 4 (ACSL4)-mediated phospholipid remodeling, enhanced fatty acid oxidation, nicotinamide adenine dinucleotide phosphate (NADPH) depletion, glutathione exhaustion, impaired glutathione peroxidase 4 (GPX4) function, and lipid peroxide amplification may collectively heighten neutrophil susceptibility to ferroptosis. We further propose a self-amplifying “ferroptotic relay,” whereby ferroptotic neutrophils release oxidized lipid mediators and damage-associated molecular patterns (DAMPs) that drive inflammatory amplification, adaptive immune modulation, and sustained neutrophil recruitment within the airway microenvironment. We also examine how this ferroptosis–metabolism axis may contribute to chronic airway inflammation, glucocorticoid resistance, and disease heterogeneity. Finally, we highlight potential therapeutic opportunities involving ferroptosis-targeting agents, metabolic interventions, and biomarker-guided patient stratification. Although direct evidence for neutrophil ferroptosis in human neutrophilic asthma is still lacking, this framework may offer a conceptual foundation for future mechanistic, translational, and clinical investigations.

## 1 Introduction

### 1.1 Clinical challenges of neutrophilic asthma

Neutrophilic asthma is a major disease subtype. It is characterized by abundant neutrophil infiltration in the airways, a predominant Type 1 T helper cells (Th1)/Type 17 T helper cells (Th17)-type immune response, and resistance to glucocorticoid therapy (1). Patients with this subtype experience more severe clinical symptoms, more frequent exacerbations, and accelerated lung function decline (2). However, no targeted therapies are currently available. Most mechanistic studies have focused on cytokine and chemokine networks, such as the regulation of neutrophil recruitment and activation by interleukin-8, interleukin-17(IL-17), and tumor necrosis factor-alpha (3). These molecular insights have not sufficiently explained why neutrophils persistently accumulate in asthmatic airways, undergo abnormal functional changes, and respond poorly to conventional anti-inflammatory treatments (4). A critical but often overlooked aspect is the interaction between cellular metabolism and cell death pathways. Specifically, it remains unclear how metabolic reprogramming within the inflammatory microenvironment directs neutrophils toward non-apoptotic death and thereby amplifies local inflammation (5).

### 1.2 Ferroptosis: an emerging mechanism from cancer to inflammatory diseases

Ferroptosis is an iron-dependent form of regulated cell death driven by lipid peroxidation (6). Its core features include intracellular accumulation of free iron, peroxidation of polyunsaturated fatty acid phospholipids, and inactivation of GPX4, which leads to the collapse of antioxidant defenses (6). Ferroptosis was initially identified as an inducible tumor suppression mechanism in cancer research (7). More recently, accumulating evidence has revealed its critical roles in inflammatory diseases (8, 9). Among immune cells, ferroptosis in macrophages exacerbates atherosclerosis and acute lung injury, whereas ferroptosis in T cells contributes to the progression of autoimmune diseases (9). Despite these advances, whether ferroptosis occurs in neutrophils—the most abundant innate immune cells with the shortest lifespan—and what pathological roles it may play in inflammation remain largely unexplored (10).

### 1.3 Knowledge gap and central thesis

Existing evidence suggests disturbed iron metabolism and elevated markers of lipid peroxidation in asthmatic airways (11). However, current support for neutrophil ferroptosis in neutrophilic asthma remains indirect. Prior investigations have largely localized ferroptotic features to airway or alveolar epithelial cells rather than to neutrophils themselves (11, 12). Although lipid peroxidation products and iron dysregulation have been detected in the asthmatic airway milieu, direct confirmation of ferroptotic neutrophils in sputum, bronchoalveolar lavage fluid, or airway tissue is still absent (13). This gap leaves unresolved whether the observed oxidative signature originates primarily from structural cells or truly reflects a neutrophil-intrinsic ferroptotic process. Consequently, the present perspective should be viewed as a hypothesis-generating framework intended to guide future mechanistic and translational studies.

Nevertheless, no study has systematically addressed whether neutrophil ferroptosis actively shapes the inflammatory microenvironment of neutrophilic asthma, nor delineated the molecular pathways and pathological consequences that would be involved (11, 12). More importantly, ferroptosis may not merely represent an endpoint of neutrophil death. Instead, ferroptosis-associated alterations in fatty acid oxidation, glutathione metabolism, mitochondrial redox balance, and NADPH availability may promote the transition of neutrophils from a resting state toward a pro-inflammatory effector phenotype. Examples of such reprogramming include mitochondrial dysfunction, remodeling of glutamine metabolism, and altered activity of NADPH oxidase (14, 15).

In the present perspective, the term “ferroptosis-metabolism axis” refers to a bidirectional regulatory network linking ferroptotic signaling with metabolic substrate availability, lipid remodeling, mitochondrial redox homeostasis, and inflammatory propagation (16, 17). Rather than functioning as a simple linear cascade, this proposed axis may operate as a dynamic and self-amplifying circuit, in which metabolic dysfunction increases susceptibility to ferroptosis, while ferroptosis-associated oxidative damage further reshapes cellular metabolism and inflammatory signaling (18, 19). Accordingly, the relationship between ferroptosis and metabolic reprogramming is unlikely to be strictly unidirectional. Instead, reciprocal interactions between redox imbalance, lipid peroxidation, mitochondrial dysfunction, and inflammatory signaling may collectively sustain chronic neutrophilic airway inflammation (13, 20).

**Figure 1:**
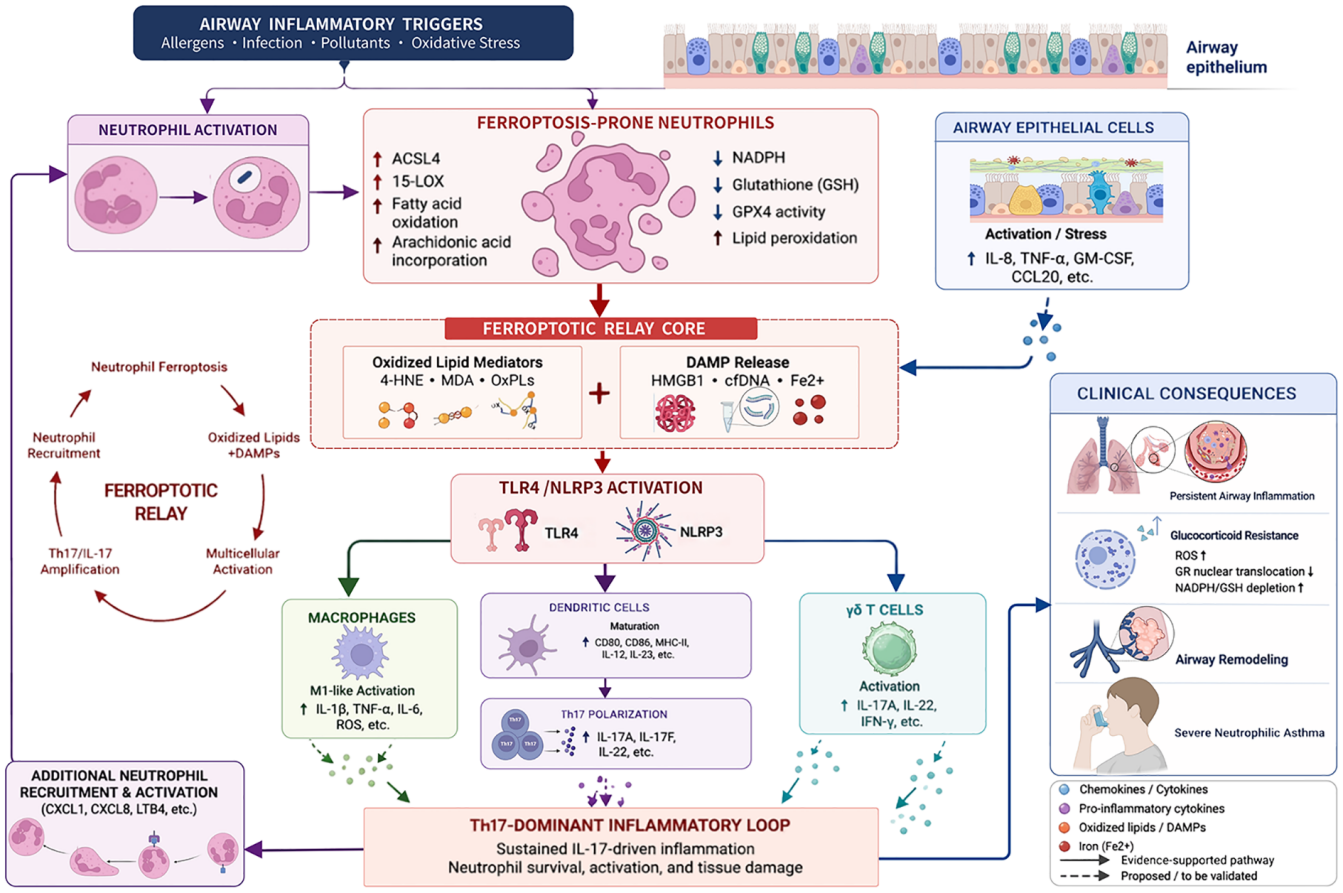
Proposed multicellular ferroptotic relay driving immunometabolic amplification in neutrophilic asthma. Schematic of the ferroptotic relay in neutrophilic asthma. Activated neutrophils undergoACSL4-driven ferroptosis, releasing DAMPs and oxidized lipids that activate TLR4/NLRP3 signaling in airway cells. This fuels Th17 inflammation and further neutrophil recruitment, establishing a self-amplifying loop that promotes glucocorticoid resistance and disease persistence.

This perspective article proposes that neutrophil ferroptosis may remodel the immuno-metabolic microenvironment of asthmatic airways by releasing lipid peroxidation products and DAMPs. Consequently, it may amplify Th1/Th17-type inflammation and potentially contribute to glucocorticoid resistance. Elucidating this mechanism may reveal novel diagnostic biomarkers and therapeutic targets for neutrophilic asthma. Recently, a review discussed the broader ferroptosis-immune-metabolic axis across asthma endotypes and its potential translational implications (13). In contrast, the present perspective specifically emphasizes neutrophil-centered ferroptotic metabolic reprogramming. It proposes a self-amplifying inflammatory feedback model, termed the “ferroptotic relay”. It also highlights the potential link between ferroptosis-associated metabolic dysregulation and glucocorticoid resistance in neutrophilic asthma (21). Furthermore, we address unresolved questions regarding heterogeneity in neutrophil death states, including the possible interplay between ferroptosis and NETosis. The proposed multicellular ferroptotic relay model and its predicted immunometabolic interactions within the airway microenvironment are summarized in **Figure 1**.

The scope and limitations of this perspective merit explicit consideration. At present, direct evidence for ferroptotic neutrophils in human neutrophilic asthma remains unavailable, and much of the current framework is extrapolated from studies of other inflammatory diseases or non-neutrophil airway cell types. Accordingly, several proposed mechanisms—including the ferroptotic relay, the interplay between ferroptosis and NETosis, and the role of ferroptosis-related metabolic dysregulation in glucocorticoid resistance—should be viewed as testable hypotheses rather than established facts. The aim of this perspective article is not to offer definitive mechanistic conclusions, but to integrate emerging evidence into a conceptual framework that may guide future mechanistic, translational, and clinical investigations.

## 2 Ferroptosis in neutrophils: molecular basis and regulatory features

### 2.1 Susceptibility of neutrophils to ferroptosis

Neutrophils have a molecular makeup that makes them prone to ferroptosis. They constitutively express high levels of two key enzymes: ACSL4 and lipoxygenase (22, 23). Both enzymes promote the incorporation and peroxidation of polyunsaturated fatty acids (PUFAs) into membrane lipids (23). At the same time, neutrophils have limited reserves of GPX4 and a weak capacity for glutathione synthesis (24, 25). This combination-abundant pro-ferroptotic enzymes together with weak antioxidant defenses-lowers the threshold for activating ferroptosis. Therefore, in inflammatory conditions such as asthma, where local iron levels and oxidative stress are elevated, neutrophils may theoretically become more susceptible to ferroptosis (16). However, it should be noted that this study primarily summarized the emerging biology of neutrophil ferroptosis across inflammatory contexts and did not directly demonstrate neutrophil ferroptosis in asthma itself. This process may in turn shape the progression of airway inflammation.

### 2.2 Interplay between ferroptosis and NETosis

Ferroptosis and NETosis are two distinct forms of regulated neutrophil death (26). They share some upstream signals, including the production of reactive oxygen species and the occurrence of lipid peroxidation (27). However, their execution mechanisms are fundamentally different. Ferroptosis is driven by progressive membrane damage caused by lipid peroxidation (6, 28). In contrast, NETosis involves chromatin decondensation and the release of neutrophil extracellular traps (NETs) without early membrane rupture (29). A key unanswered question is whether ferroptosis acts as an upstream trigger for NETosis under specific inflammatory conditions, or whether it operates independently to promote sterile inflammation. Early evidence suggests that lipid peroxidation products may disrupt the nuclear envelope, thereby facilitating the release of chromatin (27). Conversely, ferroptosis can also occur without NETs formation, contributing to sterile inflammation through the release of DAMPs (6, 27).

At present, however, the temporal and mechanistic relationship between ferroptosis and NETosis remains poorly defined (21). One possibility is that excessive lipid peroxidation destabilizes intracellular membranes and lowers the threshold for chromatin extrusion during NETosis. Alternatively, NET-associated myeloperoxidase and reactive oxygen species may further amplify lipid peroxidation, thereby promoting ferroptotic signaling in neighboring neutrophils or surrounding airway cells (13, 23). It is also plausible that ferroptosis and NETosis preferentially occur in distinct neutrophil subsets or dominate during different stages of airway inflammation, reflecting substantial cellular and temporal heterogeneity within the inflammatory microenvironment. Resolving these unresolved interactions will likely require advanced experimental approaches, including single-cell multi-omics, lineage-tracing strategies, and live-cell imaging systems capable of simultaneously tracking ferroptotic and NETotic signatures in real time. Clarifying the interplay between ferroptosis and NETosis may improve understanding of neutrophil functional diversity and help explain how distinct neutrophil death programs contribute to persistent airway inflammation and disease heterogeneity in neutrophilic asthma (13, 21, 23).

### 2.3 Metabolic reprogramming of neutrophils under ferroptosis regulation

The induction of ferroptosis in neutrophils is closely linked to changes in cellular metabolism (6). Regarding lipid metabolism, neutrophils shift away from oxidative phosphorylation and toward increased fatty acid oxidation (6). This shift makes more PUFAs available as substrates for peroxidation, creating a positive feedback loop that amplifies ferroptotic signals (30). At the same time, mitochondrial dysfunction becomes a central feature (30, 31). It is marked by impaired electron transport chain activity, reduced adenosine triphosphate production, and altered levels of tricarboxylic acid cycle intermediates (31). For instance, the accumulation of succinate coupled with the depletion of glutathione may further increase the sensitivity of neutrophils to ferroptosis (32, 33).

Mechanistically, increased ACSL4 activity may promote the incorporation of arachidonic acid and other polyunsaturated fatty acids into membrane phospholipids, thereby expanding the substrate pool available for lipid peroxidation (34). Concurrently, enhanced fatty acid oxidation and mitochondrial dysfunction may accelerate intracellular NADPH consumption and deplete glutathione reserves, thereby weakening the GPX4-dependent antioxidant defense system (13). The resulting accumulation of lipid peroxides may further disrupt membrane integrity and facilitate the release of DAMPs (13, 23). Collectively, these interconnected events may establish a self-amplifying feedback loop that reinforces both inflammatory signaling and ferroptotic susceptibility within the neutrophilic airway microenvironment.

Importantly, these metabolic disturbances are unlikely to act solely as upstream triggers or downstream consequences of ferroptosis. Rather, the present framework posits a bidirectional feedback model in which mitochondrial dysfunction, glutathione depletion, altered fatty acid metabolism, and NADPH exhaustion initially lower the threshold for ferroptotic death; reciprocally, ferroptosis-driven lipid peroxide accumulation and inflammatory signaling further aggravate metabolic dysregulation (13, 20, 23). This reciprocal amplification loop may sustain chronic inflammatory activation in neutrophilic asthma. These integrated metabolic changes may not only facilitate ferroptotic execution but also precede and contribute to the functional reprogramming of neutrophils from a quiescent state to a pro-inflammatory phenotype. Consequently, targeting these metabolic nodes offers a promising strategy to modulate neutrophil behavior in neutrophilic asthma.

### 2.4 Proposed metabolic signature of ferroptosis-prone neutrophils

Current evidence suggests that neutrophils prone to ferroptosis exhibit several characteristic metabolic alterations (13, 23). These include enhanced fatty acid oxidation, increased incorporation of PUFAs into membrane phospholipids via ACSL4, depletion of glutathione reserves, exhaustion of NADPH, accumulation of succinate, and impaired mitochondrial electron transport chain activity (20, 23). Importantly, some of these alterations may act as upstream permissive events that increase susceptibility to ferroptosis, whereas others likely represent downstream consequences of lipid peroxidation and mitochondrial dysfunction (18, 30). A major unresolved challenge is to distinguish causal metabolic drivers from secondary metabolic collapse. The proposed metabolic characteristics of ferroptosis-prone neutrophils, including key metabolic pathways, molecular alterations, directional changes, and their potential roles in ferroptotic regulation, are summarized in **Table 1**.

**Table 1:** Proposed Metabolic Signature of Ferroptosis-Prone Neutrophils.

Metabolic pathway	Key molecular changes	Direction of change	Proposed role in ferroptosis	Initiating event or downstream consequence
FAO	Increased FAO activity	↑	NADPH consumption and oxidative stress	Initiating
PUFA- phospholipid remodeling	ACSL4-mediated arachidonic acid incorporation	↑	Expands lipid peroxidation substrate pool	Initiating
Glutathione metabolism	GSH depletion	↓	Weakens antioxidant defense	Initiating
GPX4 activity	Functional impairment	↓	Failure to detoxify lipid peroxides	Core ferroptotic switch
NADPH pool	Consumption/exhaustion	↓	Limits GPX4 regeneration	Initiating
Mitochondrial respiration	ETC dysfunction	↓	Promotes ROS generation	Bidirectional
TCA cycle	Succinate accumulation	↑	Enhances inflammatory signaling	Bidirectional
Lipid peroxide burden	4-HNE, MDA accumulation	↑	Membrane damage and DAMPs release	Downstream
Cellular ATP	Energy depletion	↓	Cellular dysfunction	Downstream
DAMPs release	HMGB1, cfDNA, Fe²^+^ release	↑	Amplifies inflammation	Downstream

↑ indicates increase; ↓ indicates decrease; ACSL4, Acyl-CoA Synthetase Long-Chain Family Member 4; ATP, Adenosine Triphosphate; cfDNA, Cell-Free DNA; DAMPs, Damage-Associated Molecular Patterns; ETC, Electron Transport Chain; FAO, Fatty Acid Oxidation; Fe²^+^, Ferrous Iron; GPX4, Glutathione Peroxidase 4; GSH, Reduced Glutathione; HMGB1, High-Mobility Group Box 1; MDA, Malondialdehyde; NADPH, Nicotinamide Adenine Dinucleotide Phosphate; PUFA, Polyunsaturated Fatty Acid; ROS, Reactive Oxygen Species; TCA cycle, Tricarboxylic Acid Cycle.

### 2.5 Proposed organizational structure of the ferroptosis–metabolism axis

**Figure 2:**
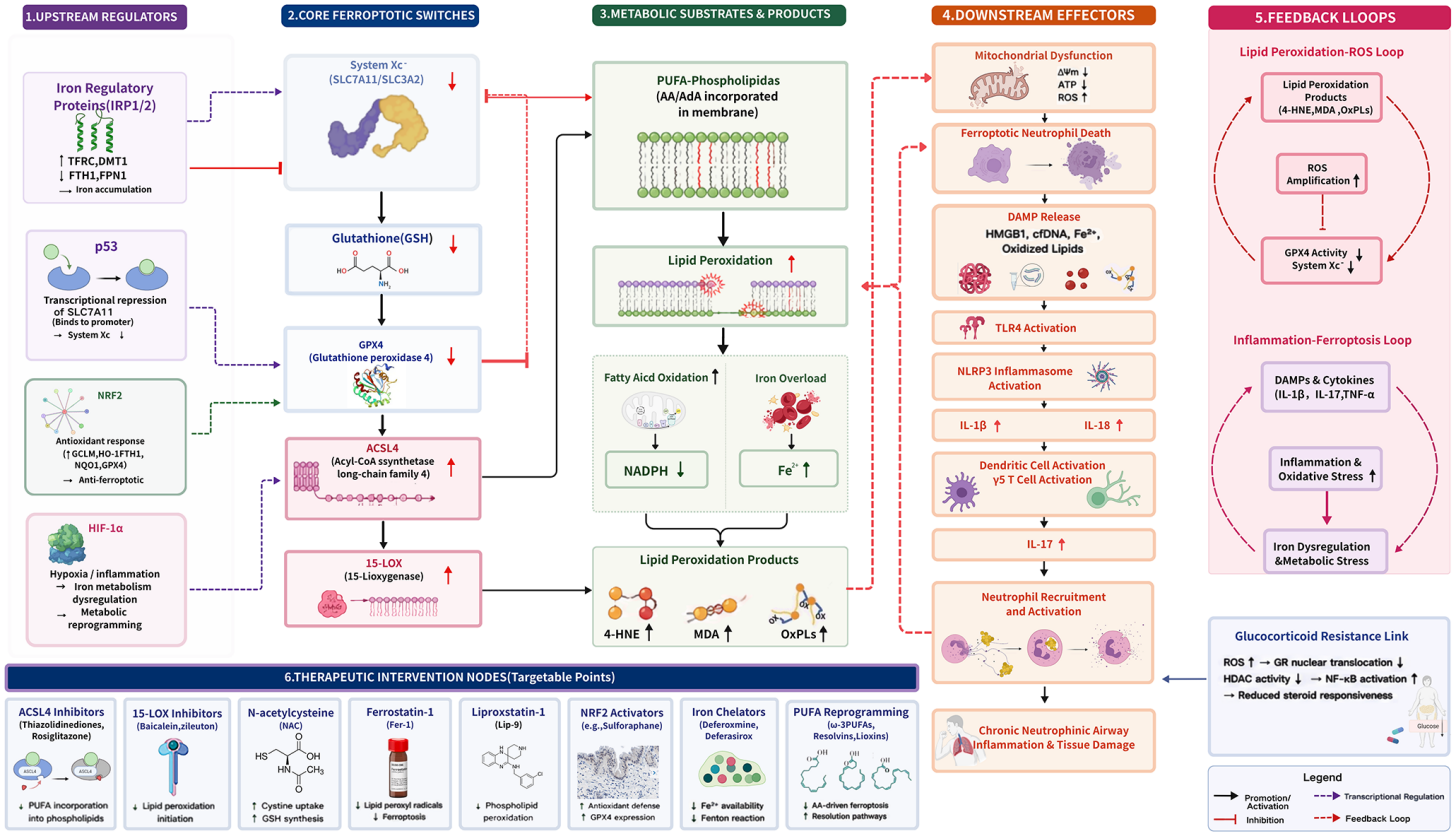
Integrated circuit diagram of the proposed ferroptosis - metabolism axis in neutrophilic asthma. Integrated circuit model illustrating upstream regulators, core ferroptotic switches, metabolic substrates and products, downstream inflammatory effectors, feedback amplification loops, and therapeutic intervention nodes within the proposed ferroptosis-metabolism axis that may contribute to persistent neutrophilic airway inflammation and glucocorticoid resistance.

The proposed ferroptosis–metabolism axis can be conceptually organized into several interconnected regulatory layers. Upstream regulatory signals may include iron regulatory proteins, p53, NRF2, and hypoxia-inducible factor-1α, all of which influence redox balance and ferroptotic susceptibility (18, 35). Core ferroptotic switches include GPX4, ACSL4, 15-lipoxygenase, and system Xc^-^, which collectively regulate PUFA-phospholipid peroxidation and antioxidant defense capacity (30). Major metabolic substrates and products include PUFA-containing phospholipids, glutathione, NADPH, Fe²^+^, 4-hydroxynonenal, and malondialdehyde (13, 18). Downstream consequences may involve mitochondrial membrane potential collapse, adenosine triphosphate depletion, release of DAMPs, and activation of inflammatory pathways such as Toll-like receptor 4/NLRP3 signaling (6, 20, 24). Importantly, several positive feedback mechanisms may further amplify this circuit, including lipid peroxide-mediated suppression of antioxidant systems and propagation of inflammatory oxidative stress to neighboring cells (20, 30). These integrated molecular interactions and feedback loops are summarized as a testable ferroptosis–metabolism circuit in **Figure 2**.

## 3 Mechanisms by which ferroptosis-associated lipid peroxidation and metabolic dysregulation shapes the inflammatory microenvironment

### 3.1 Pro-inflammatory lipid mediator storm

Ferroptotic neutrophils release oxidized lipid species, such as 4-hydroxynonenal and malondialdehyde (36). These lipids are not merely passive byproducts of cell death. Instead, they may function as bioactive mediators that propagate inflammatory signaling by activating nearby epithelial cells and macrophages (37). Mechanistically, oxidized lipid species initiate pro-inflammatory signaling pathways involving Toll-like receptor 4/NLRP3 activation, thereby enhancing the production of downstream inflammatory mediators (38–40). As a result, this lipid−driven cascade generates a surge of inflammatory signals that strongly remodels the airway environment toward a lasting pro-inflammatory state (**Table 2**).

**Table 2:** Key components of ferroptosis-driven inflammatory remodeling in neutrophilic asthma.

Module	Key molecules	Mechanism	Functional outcome	Clinical implication
Ferroptosis core	GPX4, ACSL4, LOX	Lipid peroxidation	Cell death	Therapeutic targets
Lipid mediators	MDA, 4-HNE	TLR4/NLRP3 activation	Cytokine storm	Biomarkers
DAMPs	HMGB1, DNA, Fe^2+^	Immune activation	Neutrophil recruitment	Anti-inflammatory targets
Metabolic shift	PUFA metabolism, mitochondria	Redox imbalance	Pro-inflammatory phenotype	Metabolic therapy
Adaptive immunity	IL-17, Th17	DC reprogramming	Chronic inflammation	Biologic therapy
Clinical markers	MDA, 8-iso-PGF2α, GPX4	Reflect ferroptosis	Patient stratification	Precision medicine

GPX4, glutathione peroxidase 4; ACSL4, acyl-CoA synthetase long-chain family member 4; LOX, lipoxygenase; PUFA, polyunsaturated fatty acids; MDA, malondialdehyde; 4-HNE, 4-hydroxynonenal; 8-iso-PGF2α, 8-iso-prostaglandin F2α; TLR4, Toll-like receptor 4; NLRP3, NOD-like receptor family pyrin domain containing 3; IL-17, interleukin-17; DAMPs, damage-associated molecular patterns; HMGB1, high mobility group box 1; DNA, deoxyribonucleic acid; Fe²^+^, ferrous iron; DC, dendritic cell; Th17, T helper 17 cells.

### 3.2 Positive feedback loop between neutrophil death and inflammation

Besides releasing oxidized lipids, ferroptotic neutrophils also give off various DAMPs, such as high-mobility group box 1, cell-free DNA, and free iron (13). These DAMPs act as alarm signals that draw more neutrophils to the inflamed site and activate them (41). Once recruited, these fresh neutrophils remain vulnerable to ferroptosis because they share the same metabolic weaknesses described earlier (42). Collectively, these processes may establish a self-amplifying inflammatory feedback loop that perpetuates neutrophilic airway inflammation. This cycle can keep airway inflammation going even without ongoing external triggers (13, 43, 44). Breaking this positive feedback loop may therefore provide a new way to stop persistent neutrophilic inflammation in asthma. Importantly, this proposed feedback amplification model is conceptually distinct from the initial inflammatory signaling events. While early lipid peroxidation products may trigger inflammatory activation, the subsequent persistence of ferroptosis-associated danger signaling likely contributes to the maintenance of chronic inflammation and its propagation across tissues.

### 3.3 Indirect regulation of adaptive immunity

Neutrophil ferroptosis does not only affect innate immunity; it also indirectly shapes adaptive immune responses (30, 45). One important mechanism is the reprogramming of dendritic cell maturation (46). Lipid peroxidation products and DAMPs from ferroptotic neutrophils change the local cytokine environment (47). This change may potentially favor dendritic cell phenotypes that support Th17 differentiation under certain inflammatory conditions (48). As a result, the production of IL-17 increases (49). IL-17 is a key driver of neutrophilic inflammation (49). In addition, neutrophil ferroptosis may affect γδ T cells, a group of innate-like T lymphocytes that provide an early source of IL-17 (50). Early evidence suggests a possible “neutrophil-γδ T cell axis,” through which signals from ferroptotic neutrophils boost IL-17 production (51). Through these pathways, ferroptosis-associated lipid peroxide accumulation, redox imbalance, and altered neutrophil metabolic signaling may contribute to shaping adaptive immunity and potentially support the Th17-biased immune environment observed in neutrophilic asthma (13, 24, 52). However, direct evidence remains unavailable to show that ferroptotic neutrophils specifically drive Th17 polarization in asthma (1, 20). It is also unclear whether these neutrophils preferentially promote Th17 responses over broader Th1 or Th2 immune activation. This unresolved question warrants dedicated experimental investigation.

## 4 Clinical and translational implications: targeting ferroptosis-metabolism axis

**Figure 3:**
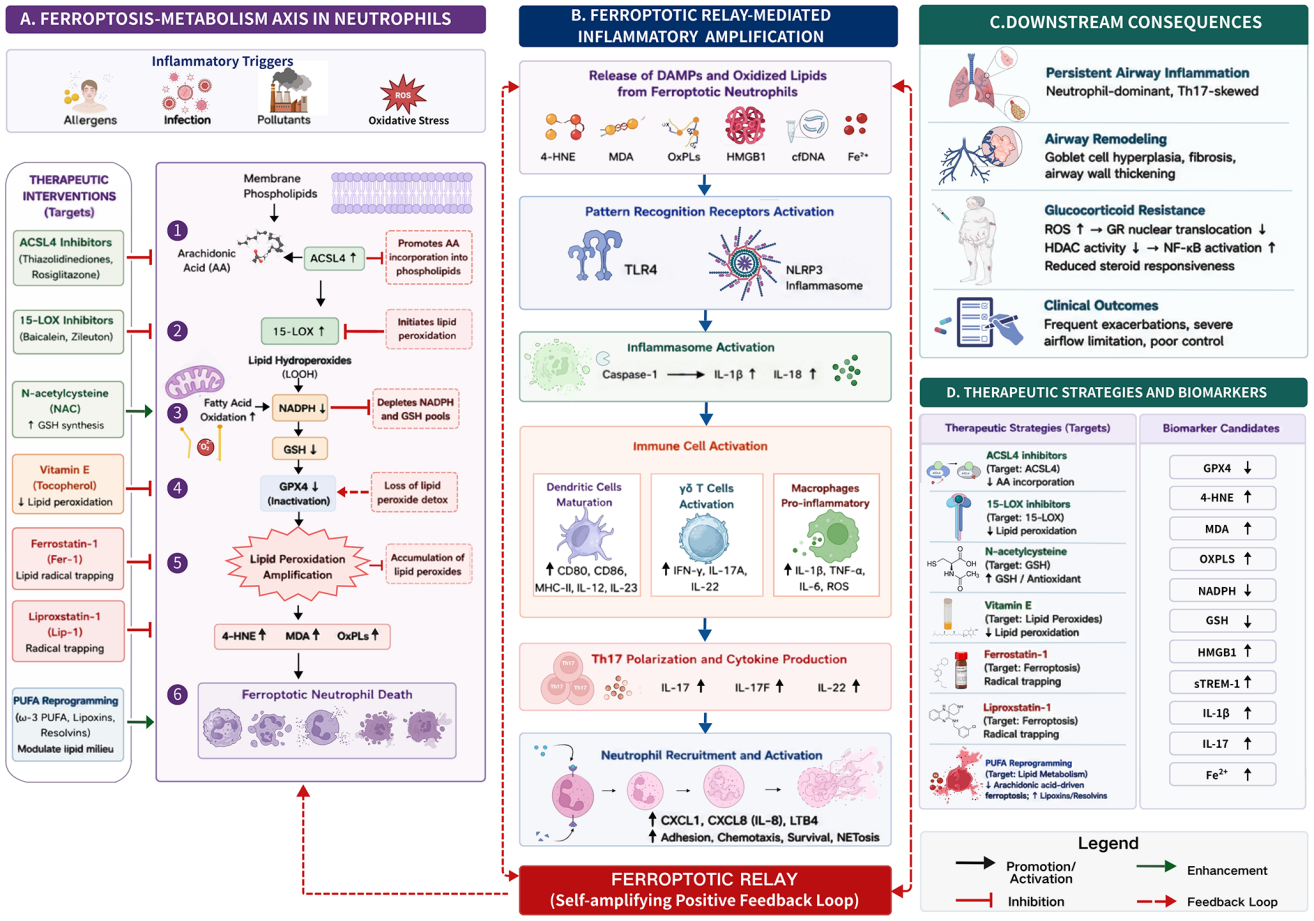
Therapeutic targeting of the ferroptosis-metabolism axis in neutrophilic asthma. Schematic overview of the ferroptosis-metabolism axis in neutrophilic asthma, highlighting key molecular regulators, ferroptotic relay-mediated inflammatory amplification, glucocorticoid resistance, biomarker candidates, and potential therapeutic intervention targets.

Potential therapeutic intervention points across the ferroptosis–metabolism axis are illustrated in **Figure 3**.

### 4.1 Re-evaluation of existing drugs

Recognizing ferroptosis as a driver of neutrophilic asthma calls for a fresh look at existing drugs. Ferroptosis inhibitors like ferrostatin-1 and liproxstatin-1 have shown protective effects in preclinical models of acute lung injury and asthma (53, 54). They work by limiting lipid peroxidation and preserving GPX4 activity (30). However, their effectiveness specifically in neutrophilic asthma has not yet been tested in a systematic way. Other agents that affect the antioxidant system, such as N-acetylcysteine and vitamin E, have been tried as add-on treatments for asthma (55). N-acetylcysteine helps produce glutathione, while vitamin E stops lipid peroxidation chain reactions (56). But their clinical benefits have been inconsistent. One reason is that they do not directly block the pro-ferroptotic enzymes (for example, ACSL4 and lipoxygenase) that drive lipid peroxidation in neutrophils.

One possible mechanism linking ferroptosis to glucocorticoid resistance involves persistent oxidative stress and excessive lipid peroxidation that overwhelm corticosteroid-responsive anti-inflammatory pathways. Sustained accumulation of reactive oxygen species may impair glucocorticoid receptor nuclear translocation, disrupt histone deacetylase activity, and maintain NF-κB-dependent inflammatory signaling despite corticosteroid exposure (57). In parallel, ferroptosis-associated depletion of glutathione and NADPH may further destabilize intracellular redox homeostasis, thereby reducing corticosteroid responsiveness (18, 58). These mechanisms may help explain why neutrophilic asthma frequently exhibits poor sensitivity to glucocorticoid therapy despite aggressive anti-inflammatory treatment (1, 13). However, current evidence remains indirect, and the proposed relationship between ferroptosis-associated metabolic dysregulation and glucocorticoid resistance should presently be regarded as a hypothesis requiring direct experimental validation in neutrophilic asthma models and patient-derived samples.

Therefore, although current antioxidant and ferroptosis-targeting agents show therapeutic promise, their limitations highlight the need for more selective and mechanistically informed interventions capable of modulating ferroptosis-related metabolic pathways in airway neutrophils.

### 4.2 Metabolic intervention strategies

A more targeted approach is to directly block the key enzymes that start ferroptosis in neutrophils. Small-molecule inhibitors against ACSL4 and 15-lipoxygenase have already been developed and tested in other inflammatory diseases (59–61). These inhibitors could prevent the uptake and peroxidation of PUFAs in neutrophil membranes (62, 63). This would stop ferroptosis without broadly weakening the body’s antioxidant defenses (59, 62). Another promising method is to change PUFAs metabolism so that it shifts away from pro-ferroptotic pathways and toward anti-inflammatory lipid signals. For example, steering arachidonic acid metabolism toward lipoxins or resolvins instead of leukotrienes might at the same time lower ferroptosis risk and help resolve inflammation (63). Such metabolic changes could bring two benefits at once in neutrophilic asthma.

### 4.3 Biomarkers and patient stratification

Finding biomarkers linked to ferroptosis could help group patients and guide personalized care. In clinical practice, doctors can measure lipid peroxidation products like malondialdehyde and 8-iso-prostaglandin F2α in sputum or serum (64). These levels serve as indirect signs of ferroptosis in the airways. In addition, the amount of GPX4 in airway neutrophils may act as a functional biomarker (21). Patients with low GPX4 levels in their neutrophils may be more prone to ferroptosis (21). As a result, they may also be more likely to resist glucocorticoid treatment (65). At present, however, no validated quantitative threshold has been established to define when ferroptosis-associated metabolic dysregulation becomes clinically sufficient to drive steroid resistance or severe airway dysfunction (66). Several candidate indicators warrant systematic investigation, including oxidized phospholipid burden, intracellular NADPH depletion, lipid peroxide accumulation, and mitochondrial membrane potential collapse (13, 18, 67). Longitudinal studies that integrate metabolomic profiling, lung function trajectories, corticosteroid responsiveness, and airway inflammatory phenotyping will be essential to determine whether specific metabolic flux signatures can reliably distinguish treatment-responsive from treatment-resistant neutrophilic asthma. Identifying these biomarkers could help identify which patients would gain the most from therapies that target ferroptosis. This would move asthma care beyond the current one-size-fits-all approach.

## 5 Future research directions and key questions

### 5.1 Dynamic imaging and single-cell metabolomics

A key research goal is to visualize and measure neutrophil ferroptosis in real time inside the asthmatic airway. Dynamic imaging methods, such as intravital microscopy, could help track iron buildup, lipid peroxidation, and membrane damage in neutrophils as inflammation unfolds (68). Single-cell metabolomics can add to this by revealing metabolic differences among neutrophils in different asthma subtypes (69, 70). Not all neutrophils in an inflamed airway are equally likely to undergo ferroptosis (71). Some may have metabolic traits that make them more vulnerable, while others may be protected. Mapping this heterogeneity at single-cell resolution is therefore essential for identifying which neutrophil populations preferentially contribute to ferroptosis-associated inflammation and whether these patterns differ among neutrophilic, eosinophilic, and mixed granulocytic asthma phenotypes.

Importantly, despite growing interest in ferroptosis-associated immunometabolism, substantial knowledge gaps remain regarding how ferroptotic susceptibility and metabolic reprogramming differ across individual airway cell populations (13). Beyond neutrophils alone, epithelial cells, macrophages, fibroblasts, and other airway-resident cells may exhibit distinct ferroptosis-associated metabolic states depending on their antioxidant capacity, mitochondrial dependency, and lipid metabolic characteristics (13, 72). Future studies integrating single-cell metabolomics, single-cell transcriptomics, and spatially resolved imaging technologies may therefore help define cell-type-specific ferroptotic signatures while simultaneously uncovering spatial metabolic interactions within the inflammatory airway microenvironment. Such approaches could further clarify whether ferroptosis-associated metabolic remodeling occurs in coordinated multicellular networks rather than as an isolated neutrophil-intrinsic process.

### 5.2 Intercellular metabolic communication

Beyond what happens inside neutrophils, future work should explore how ferroptotic neutrophils communicate with nearby structural cells, including airway epithelial cells and smooth muscle cells. Dying neutrophils release not only DAMPs but also oxidized lipids carried within extracellular vesicles (13). These vesicles may function as carriers that transport pro-inflammatory lipid mediators to distant sites within the airway tissue, thereby propagating inflammation even in the absence of direct cell-to-cell contact (73).

Importantly, the ferroptosis-metabolism axis is unlikely to function uniformly across all airway cell populations. Neutrophils, epithelial cells, macrophages, fibroblasts, and airway smooth muscle cells differ substantially in antioxidant capacity, mitochondrial dependency, lipid metabolic profiles, and iron-handling mechanisms (13, 74). Consequently, ferroptosis-associated metabolic signaling may generate distinct biological responses depending on the cellular context. In some cell populations, ferroptosis-associated oxidative stress may predominantly amplify inflammatory signaling, whereas in others it may preferentially contribute to tissue remodeling, metabolic adaptation, or altered immune responsiveness (13, 19). Moreover, intercellular metabolic exchange within the airway microenvironment may further complicate these interactions. Future studies should therefore investigate whether the transfer of lactate, succinate, glutathione precursors, or oxidized lipid-containing vesicles between neighboring cells contributes to tissue-level inflammatory amplification and metabolic synchronization in neutrophilic asthma.

At present, however, the mechanisms governing intercellular metabolic communication within the ferroptosis-metabolism axis remain poorly understood (13, 16). In particular, it is unclear whether ferroptosis-associated metabolic signaling exerts similar or divergent effects across neutrophils, epithelial cells, macrophages, fibroblasts, and airway smooth muscle cells. Future experimental studies should therefore incorporate multicellular co-culture systems capable of modeling airway metabolic crosstalk under inflammatory conditions. In parallel, conditional knockout mouse models targeting key ferroptosis regulators—such as GPX4, ACSL4, or system Xc^-^—in specific airway cell lineages may help clarify the relative contribution of distinct cell populations to tissue-level inflammatory amplification and glucocorticoid resistance (13, 75, 76).

Understanding how ferroptotic neutrophils “instruct” resident structural and immune cells to adopt pro-inflammatory or tissue-remodeling phenotypes could provide important insights into the mechanisms underlying persistent airway inflammation and progressive tissue dysfunction (13, 77). This line of investigation may also identify novel therapeutic strategies aimed at disrupting pathological intercellular metabolic crosstalk within the asthmatic airway microenvironment.

### 5.3 Combination therapy strategies

From a treatment development viewpoint, combining ferroptosis-targeting agents with other anti-inflammatory therapies deserves attention. One promising idea is to pair ferroptosis inhibitors with biologics that block interleukin-17 or leukotriene B4. This would attack two cooperating drivers of neutrophilic inflammation at the same time (78). Such combinations might work better than either drug alone, especially in patients with severe asthma that does not respond to glucocorticoids (78). On the other hand, the idea of using ferroptosis inducers in certain asthma subtypes should be treated with care (19). While inducing ferroptosis might help when dying cells are not cleared properly or when harmful neutrophils need to be removed, it also carries real risks of unwanted tissue damage and worse inflammation (11). Therefore, any use of ferroptosis inducers would need careful, subtype-by-subtype testing, ideally guided by the biomarkers discussed earlier.

## 6 Summary

In this perspective article, we propose a hypothesis-generating framework suggesting that ACSL4-dependent phospholipid remodeling, NADPH depletion driven by fatty acid oxidation, and ferroptosis-associated lipid peroxide amplification may serve as key yet underrecognized mechanisms shaping the inflammatory microenvironment in neutrophilic asthma. Neutrophils are naturally prone to ferroptosis because they highly express ACSL4 and lipoxygenase enzymes while having limited GPX4 reserves. When neutrophils undergo ferroptosis, they release oxidized lipids and DAMPs. These processes may collectively amplify local inflammation, sustain maladaptive neutrophilic immune activation, and indirectly support Th17-skewed adaptive immune responses. This neutrophil-centered framework further extends recent discussions of the ferroptosis-immune-metabolic axis in asthma by emphasizing ferroptosis-associated feedback amplification, neutrophil functional heterogeneity, and corticosteroid-insensitive inflammatory persistence.

Within the proposed framework, glucocorticoid resistance may not simply reflect a failure of anti-inflammatory signaling. Instead, it may partially arise from persistent non-apoptotic neutrophil death that keeps inflammation going even during corticosteroid therapy. Targeting the ferroptosis-metabolism axis-for example, with small-molecule inhibitors against ACSL4 or 15-lipoxygenase, by reprogramming PUFAs metabolism, or through biomarker-guided patient selection-may open up precise treatment options for this difficult asthma subtype.

Nevertheless, important uncertainties remain regarding the cell-type specificity of ferroptosis-associated metabolic signaling and the mechanisms that coordinate intercellular metabolism within the airway microenvironment. Addressing these unresolved questions will require integrated experimental approaches that combine single-cell metabolomics, spatial profiling, multicellular co-culture systems, and lineage-specific genetic models. Such approaches may help clarify how ferroptosis-associated metabolic communication contributes to chronic airway inflammation, tissue remodeling, and corticosteroid resistance in neutrophilic asthma.

## Data Availability

The original contributions presented in the study are included in the article/supplementary material. Further inquiries can be directed to the corresponding author.
